# Exploring the “Dark Matter” of Social Interaction: Systematic Review of a Decade of Research in Spontaneous Interpersonal Coordination

**DOI:** 10.3389/fpsyg.2021.718237

**Published:** 2021-10-11

**Authors:** Julia Ayache, Andy Connor, Stefan Marks, Daria J. Kuss, Darren Rhodes, Alexander Sumich, Nadja Heym

**Affiliations:** ^1^Department of Psychology, Nottingham Trent University, Nottingham, United Kingdom; ^2^School of Future Environments, Auckland University of Technology, Auckland, New Zealand; ^3^Department of Psychology, Auckland University of Technology, Auckland, New Zealand

**Keywords:** interpersonal coordination, two-body neurosciences, systematic review, behavioral matching, interactional synchrony

## Abstract

Interpersonal coordination is a research topic that has attracted considerable attention this last decade both due to a theoretical shift from intra-individual to inter-individual processes and due to the development of new methods for recording and analyzing movements in ecological settings. Encompassing spatiotemporal behavioral matching, interpersonal coordination is considered as “social glue” due to its capacity to foster social bonding. However, the mechanisms underlying this effect are still unclear and recent findings suggest a complex picture. Goal-oriented joint action and spontaneous coordination are often conflated, making it difficult to disentangle the role of joint commitment from unconscious mutual attunement. Consequently, the goals of the present article are twofold: (1) to illustrate the rapid expansion of interpersonal coordination as a research topic and (2) to conduct a systematic review of spontaneous interpersonal coordination, summarizing its latest developments and current challenges this last decade. By applying Rapid Automatic Keyword Extraction and Latent Dirichlet Allocation algorithms, keywords were extracted from PubMed and Scopus databases revealing the large diversity of research topics associated with spontaneous interpersonal coordination. Using the same databases and the keywords “behavioral matching,” “interactional synchrony,” and “interpersonal coordination,” 1,213 articles were identified, extracted, and screened following the Preferred Reporting Items for Systematic Reviews and Meta-Analyses protocol. A total of 19 articles were selected using the following inclusion criteria: (1) dynamic and spontaneous interactions between two unacquainted individuals (2) kinematic analyses, and (3) non-clinical and non-expert adult populations. The results of this systematic review stress the proliferation of various definitions and experimental paradigms that study perceptual and/or social influences on the emergence of spontaneous interpersonal coordination. As methods and indices used to quantify interpersonal coordination differ from one study to another, it becomes difficult to establish a coherent picture. This review highlights the need to reconsider interpersonal coordination not as the pinnacle of social interactions but as a complex dynamical process that requires cautious interpretation. An interdisciplinary approach is necessary for building bridges across scattered research fields through opening a dialogue between different theoretical frameworks and consequently provides a more ecological and holistic understanding of human social cognition.

## Introduction

Studies of social interactions are situated at the crossroads of anthropology, sociology, philosophy, and psychology, resulting in a scattered vision of the phenomena occurring during information exchanges. While a global picture of the mechanisms underlying social interactions has not emerged yet, cumulative evidence observed a positive association between joint motor action and social bonding tendencies, generating an increased interest to study motor correlates of social interactions. Historically, French sociologists Emile Durkheim (1912) and Gustave Le Bon (1895) already observed “collective effervescence” when individuals come together during the practice of religious rituals and start to share the same actions and thoughts. Psychologists extended the notion of togetherness to more intimate interaction in early life (Tronick and Cohn, 1989; Maister et al., 2020) and processes occurring in group dynamics (Reinero et al., 2021). Contemporary studies investigating the emergence of interpersonal coordination have grown considerably, stressing the role played by joint motor action in the emergence of social bonding and affiliation. However, the literature is scattered across fields leading to a confused picture of the phenomenological aspects of social interaction and their related motor aspects. Recent attempts have been made to fill this gap, calling for a “two-body approach” capturing the dynamic exchange between individuals, labeled as “dark matter” of social interaction (Hari and Kujala, 2009; Dumas, 2011; Schilbach et al., 2013). Almost 10years after this call, this review aims to present the current state of the research field of spontaneous interpersonal coordination, highlighting its latest developments, challenges, and limitations.

### Interpersonal Coordination, an Ill-Defined Concept

According to Bernieri and Rosenthal (1991), interpersonal coordination can refer to behavioral matching or interactional synchrony. *Behavioral matching*, also labeled as “mimicry” or chameleon effect (Lakin et al., 2003), is characterized by imitative gestures (e.g., scratching nose, nodding head), which does not require behavioral synchronization in time. Thus, imitative gestures can appear after a lag of several seconds. In contrast, *interactional synchrony* relies on temporal matching, encompassing behavioral (e.g., gestures and speech) and physiological coupling, such as heart rate, skin conductance, or inter-brain synchronization (Dumas et al., 2010; Coutinho et al., 2019, 2021). Despite the attempt to delineate behavioral matching from interactional synchrony, the threshold separating these two concepts is not well-defined, and most studies use different time lags for assessing these phenomena (from 0.04 to 4s), leading to conflation of these two constructs (Schoenherr et al., 2019). To complicate matters further, the term synchrony is often understood as “in-phase” patterns, where individuals execute similar or symmetrical movements, whereas synchrony can also encompass “anti-phase” patterns, where individuals execute similar movements alternately (Haken et al., 1985). Additionally, interpersonal coordination can be considered as an unconscious phenomenon resulting from perceptual constraints (e.g., seeing each other) or as an explicit goal of joint action, resulting from mechanical couplings such as dancing or playing music together (Athreya et al., 2014). Consequently, goal-oriented interpersonal coordination can result from a unidirectional coupling (e.g., synchronization of movements with an external metronome) or through mutual adaptation (e.g., adjusting to each other’s movements). Taken together, these various conceptualizations of interpersonal coordination stress the lack of a clear definition, preventing the emergence of a deeper understanding of its mechanisms.

### Motor Coordination As Social Glue

Interpersonal coordination is thought to act as a social glue and has been linked to the concept of shared flow, defined as “a state of synchronized collective optimal experience” (Zumeta et al., 2016, p. 717). As such, it ranges from group dynamics to dyadic interactions, occurring in social settings such as collective sports (Zumeta et al., 2016), religious rituals (Perry et al., 2021), or aesthetic experiences (Vuoskoski and Reynolds, 2019) and is associated with pro-sociality and social bonding (for a review and meta-analysis, see Rennung and Göritz, 2016; Vicaria and Dickens, 2016). It can be induced experimentally through joint action and increase altruism and trust (Wiltermuth and Heath, 2009; Lang et al., 2017), but also reduce intergroup conflict in real or virtual settings (Hasler et al., 2014; Tamborini et al., 2018). According to Atherton et al. (2019), the mere imagination of walking in synchrony with an out-group member might be sufficient to decrease prejudice and stereotyping. Spontaneous interpersonal coordination can also be measured during naturalistic social exchanges and appears as a good indicator of the quality of the relationship between parent and infants (Feldman, 2007), teacher and students (LaFrance, 1979), and therapist and clients (Tschacher et al., 2015). Taken together, these observations highlight the positive association of spontaneous and goal-oriented interpersonal coordination with affiliative and pro-social tendencies. However, what exactly sustains this “social glue” is still unclear and heavily debated in the literature.

### Intra-Individual Mechanisms of Interpersonal Coordination

A first set of hypotheses attempt to explain the association between interpersonal coordination and social bonding based on embodiment theories, where interpersonal coordination is understood as a motor correlate of cognitive processes. One hypothesis suggests that interpersonal coordination induces a sense of self-other overlap, where participants feel “closer” or more “similar” to their partner, leading to more altruistic and pro-social tendencies (Ashton-James et al., 2007). Whilst this hypothesis is in line with theories accounting for developmental trajectories of empathy (Meltzoff and Decety, 2003), recent findings challenge this view. For example, Cross et al. (2020) suggested that interpersonal coordination is a result of categorization processes (e.g., self-identification as a group member) rather than feelings of similarity and closeness. A second hypothesis proposes reinforcement learning processes where successful interpersonal coordination provides rewarding feedback, reinforcing cooperative tendencies (Reddish et al., 2013). Therefore, shared intentionality rather than movement synchrony *per se* elicits social bonding, stressing the importance of goal-oriented motivation rather than a spontaneous process (Kirschner and Tomasello, 2010). A third hypothesis challenges this suggestion, proposing that the mere process of moving together is sufficient for eliciting rewarding responses as moving in synchrony reduces environmental uncertainty by increasing movements’ predictability through mirroring processes (Hove and Risen, 2009; Hoehl et al., 2020). In line with this, developmental studies support the role of synchronous bouncing movements in increasing helping behaviors, where shared intentionality is absent (Cirelli et al., 2014). To summarize, a robust and positive association has been observed between interpersonal coordination and pro-social tendencies, although the mechanisms underlying this association are still debated.

### Motivational and Dynamical Inter-Individual Processes

Recent observations stress the role of contextual and social cues that influence interpersonal coordination (Heyes, 2018; Levy and Bader, 2020). In-group membership or preexisting acquaintance (e.g., friendship) and affective bonds (e.g., romantic partners) are known to increase interpersonal coordination (Coutinho et al., 2019, 2021), while lack of relatedness such as out-group membership tends to decrease behavioral synchrony (Miles et al., 2011). Moreover, deficits in interpersonal coordination might be the results of inter-individual processes rather than sole intra-individual mechanisms, stressing the need to go beyond neurocognitive explanations (Bolis et al., 2017). For example, non-clinical participants tend to display lower tendencies for interpersonal coordination when they believe they interact with patients suffering from affective disorder or schizophrenia (Rainteau et al., 2020). Therefore, it is crucial to consider motivational aspects in interpersonal coordination that facilitate affiliation. These processes might also explain its detrimental outcomes. Interpersonal coordination is not always beneficial and can increase aggression among football supporters (Bensimon and Bodner, 2011), or compliance with destructive obedience observed in crowds or military contexts (Wiltermuth, 2012a,b). Taken together, these observations highlight the need to shift from an intra-individual to inter-individual perspective when investigating interpersonal coordination. This shift has been suggested by the theoretical framework of coordination dynamics (Kelso, 1995), proposing that interpersonal coordination is a phenomenon driven by dynamical principles of self-organization observed in living and nonliving organisms, such as the spontaneous coordination of bird flocks or a fish school. According to this theoretical viewpoint, interpersonal coordination is a pattern arising from dynamic exchanges between biologically rhythmic units through mechanical or informational coupling (Schmidt et al., 2011). Coordination dynamics provide a theoretical framework to study social interaction, understood as any situation involving perceptual and/or linguistic information exchanges between two or more individuals. This perspective might fulfil the current pitfalls observed in intra-individual models that are insufficient to take proper account of dynamic interpersonal coordination—a phenomenon that cannot be reduced to properties of each system independently (Marmelat and Delignières, 2012). This shift from studying intra-individual processes to dynamical inter-individual systems is in substance the call made by the “two-body approach,” encouraging new experimental paradigms to unravel the mechanisms underlying social interaction (Hari and Kujala, 2009; Dumas, 2011; Schilbach et al., 2013).

### Objectives of the Present Review

Almost 10years since the call for a “two-body approach,” the present systematic review aims to outline the current state of the research field of interpersonal coordination, highlighting its latest developments, challenges, and limitations. New methods such as motion energy analysis (Ramseyer, 2020) or motion tracking using Kinect (Papadopoulos et al., 2014) allow the simultaneous recording of dyads using low-cost experimental designs. Consequently, the research field of interpersonal coordination has grown considerably, providing new insights, but preventing the emergence of a coherent picture. The first goal of this article is to present an exploratory analysis underlying the richness and diversity of the themes currently addressed by interpersonal coordination research. The second aim is to deliver a systematic review of the literature, focusing on spontaneous interpersonal coordination rather than intentional and goal-oriented coordination. The research field associated with goal-oriented coordination embraces another branch of the literature concerning joint action, addressing the cognitive mechanisms underlying mutual coordination (Sebanz and Knoblich, 2021). Interpersonal coordination is distinguished from joint action to assess the impact of incidental movement coordination without shared intentionality, anticipation, or explicit instructions for movement synchronization. Although joint action research overlaps with interpersonal coordination, and the two are often combined in the same experimental setting, it includes a more extensive range of actions that require shared goal-oriented motor coordination, such as synchronizing to the same external stimuli (e.g., metronome) or producing a specific sequence of gestures (e.g., moving an object from one location to another) rather than spontaneous spatiotemporal behavioral matching. Finally, previous studies investigated the emergence of physiological or facial signatures of mutual attunement among pre-acquainted individuals such as romantic partners (Coutinho et al., 2019, 2021), parent–infant interactions (Feldman, 2007) and in collective activities such as musical ensembles (Keller, 2014) or sports (Zumeta et al., 2016). Consequently, this review will focus on the motor correlates of spontaneous interpersonal coordination in dyads among strangers to better understand the processes involved in impromptu interactions in daily life.

## Materials and Methods

First, an exploratory search conducted using the R package “RISmed” (Kovalchik, 2015) extracted contents from the PubMed database of published journal articles, allowing to identify articles containing the keyword “interpersonal coordination” in articles published from 1970 to 2020. Second, a “naive” search conducted using the R package “litsearchr” (Grames et al., 2019) identified potential additional keywords associated with interpersonal coordination. For this purpose, three datasets were extracted from PubMed using the following set of keywords: “spontaneous interpersonal coordination,” “spontaneous behavioral matching,” “spontaneous interactional synchrony.” Automated scripts removed duplicate content, and the Rapid Automatic Keyword Extraction (RAKE) algorithm identified and extracted keywords from the preexisting scientific literature. As most of the keywords found were irrelevant for the present literature, a final set of keywords was created and combined in the following Boolean search: ((spontaneous) AND (interpersonal) AND (behavioral matching) OR (interactional synchrony) OR (interpersonal coordination). Articles extracted were indexed in PubMed and Scopus (Psychology subject area only) databases corresponding with this set of keywords. Duplicate content and articles published before 2010 or after 2020 were automatically removed. The Latent Dirichlet Allocation (LDA) algorithm classified articles sharing a similar set of keywords using the R package “revtools” (Westgate, 2019). Through an iterative process (10k steps), the LDA identified five main topics. Finally, the articles were screened using the Preferred Reporting Items for Systematic Reviews and Meta-Analyses (PRISMA) protocol (Moher et al., 2015). The following inclusion/exclusion criteria were applied to select studies involving only (1) dynamic and spontaneous interactions between two unacquainted individuals (2) kinematic/movements analysis, and (3) non-clinical and non-expert adult populations. Consequently, articles rejected involved those including clinical populations (e.g., autistic, motor coordination or affective disorders), experts (e.g., dancers, musicians, sport), acquainted individuals (e.g., friends, romantic partners), children or adult-children dyads, groups (e.g., triads or larger group) and pre-recorded interactions (e.g., video recording, pictures). Additionally, purely computational or methodological investigations were also automatically rejected. Lastly, the present review focuses on body motor correlates of social interaction rather than physiological or facial signature of mutual attunement. Consequently, investigations of interpersonal physiological synchrony (e.g., hyperscanning), facial expression or mutual gazes were also automatically rejected. R scripts and datasets are available online in the following Open Science Framework repository.^1^

## Results

### Exploratory Research

**Figure 1:**
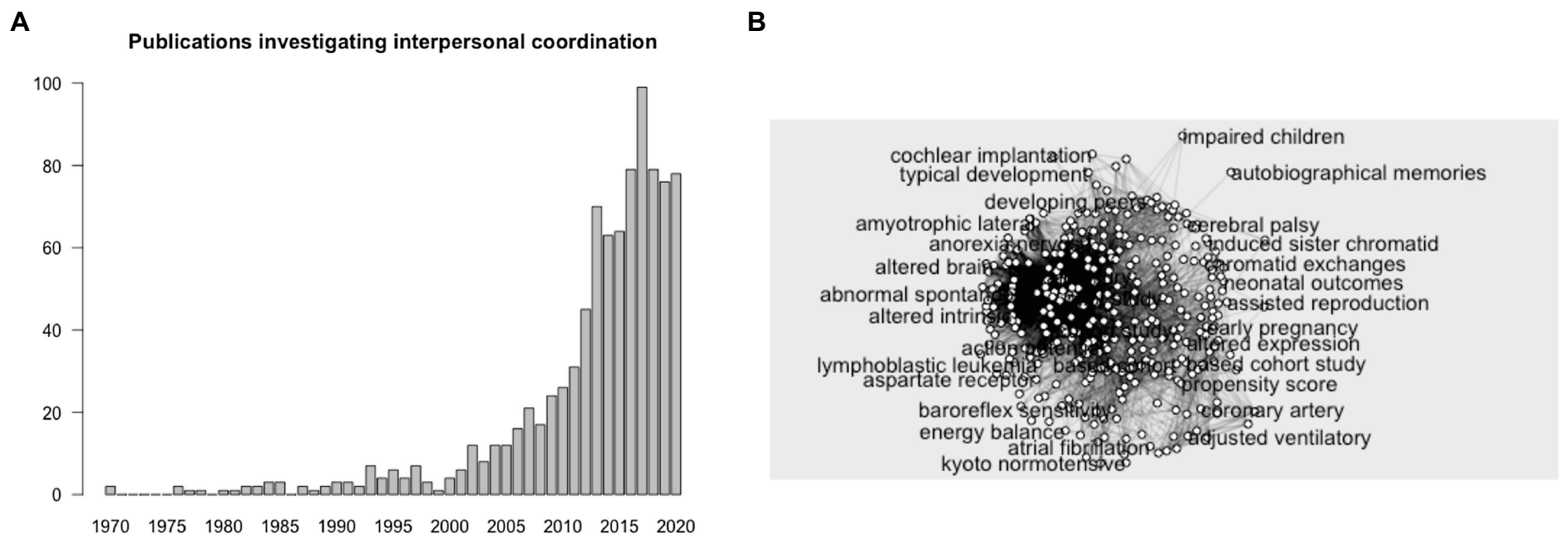
Histogram of publications registered on PubMed associated with keyword “interpersonal coordination” from 1970 to 2020 **(A)** and keywords network associated with interpersonal coordination extracted from PubMed **(B)**.

First, confirming the expectations found in the preexisting literature, the exploratory research on PubMed revealed a sharp increase in publications associated with the keywords “interpersonal coordination,” with a total of 933 publications cumulated from 1970 until 2020 and 99 publications recorded for the year 2017 (see Figure 1A). Second, the naive research and keywords network analysis revealed a broad spectrum of topics associated with interpersonal coordination. Visual inspection of the network revealed the inadequacy of the keywords suggested by the RAKE algorithm (see Figure 1B). Most of the keywords were irrelevant (e.g., hearing loss, fetal death, alcohol drinking) or targeting specific components of interpersonal coordination (e.g., facial mimicry, facial expression) or populations (e.g., autism spectrum, bipolar disorder). Consequently, this systematic review did not retain these keywords for the database search.

### Thematic Analysis

**Figure 2:**
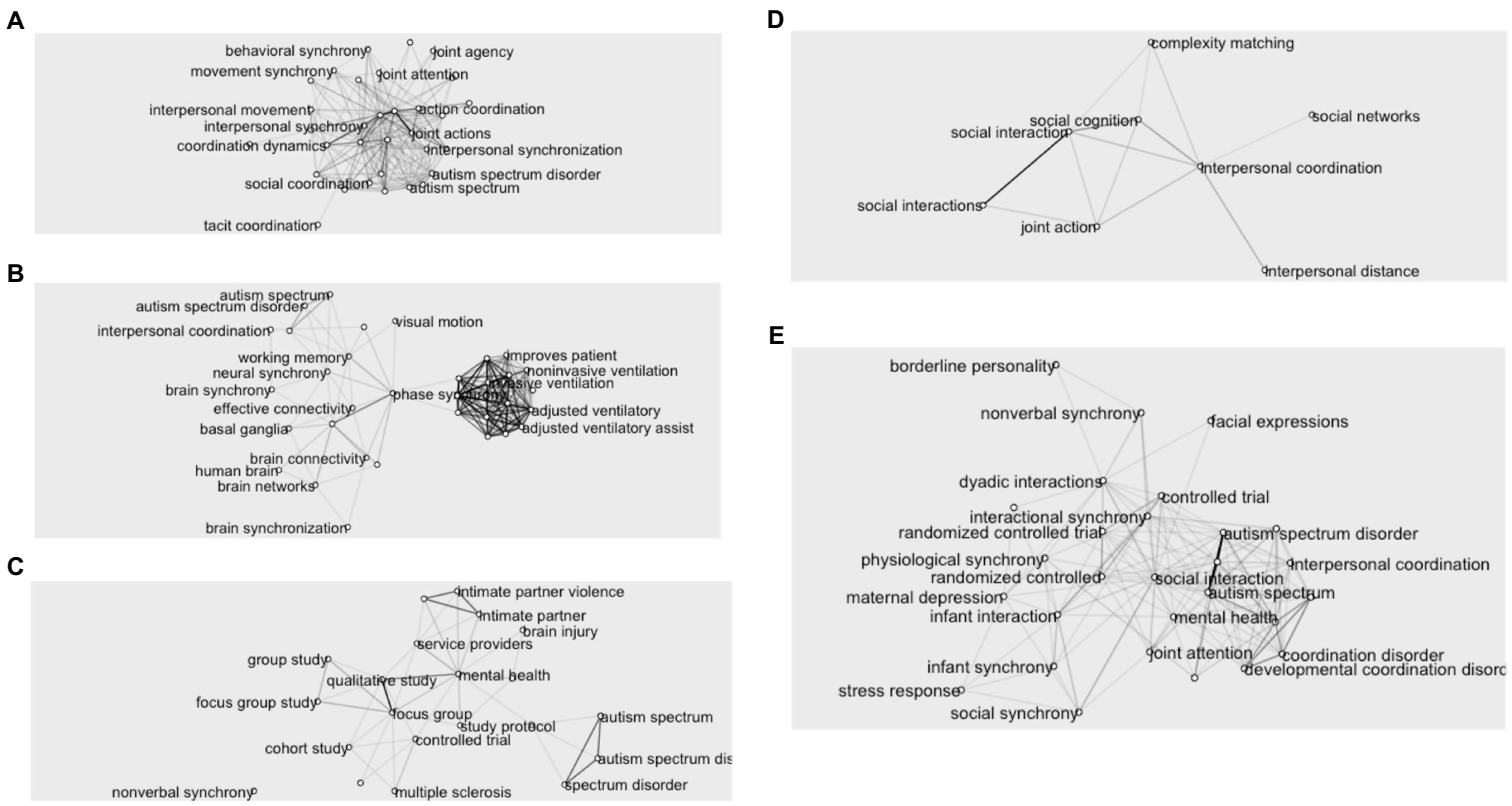
Keywords network associated with the five topics **(A-E)**.

Using the set of keywords identified within the preexisting literature (e.g., ((((spontaneous) AND (interpersonal)) AND (behavioral matching)) OR (interactional synchrony)) OR (interpersonal coordination), 1,346 articles were extracted from PubMed (774) and Scopus (572). Duplicates (129) or articles published before 2010 to after 2020 (4) were removed from the selection, resulting in a final selection of 1,213 articles. Thematic analysis based on title and abstract content conducted with LDA identified five main topics (see keywords network for each topic in Figure 2): (A) 297 articles on motor coordination in joint action (e.g., social coordination, coordination dynamics); (B) 220 articles on brain and neural synchrony in pathological and healthy populations (e.g., autism, brain connectivity); (C) 231 on patient care and community coordination (e.g., focus-group, qualitative study); (D) 246 on group dynamics (e.g., social networks, complexity matching); and (E) 219 on behavioral and physiological attunement within parental and mother–children dyads (e.g., infant synchrony, maternal depression).

### Systematic Review

**Figure 3:**
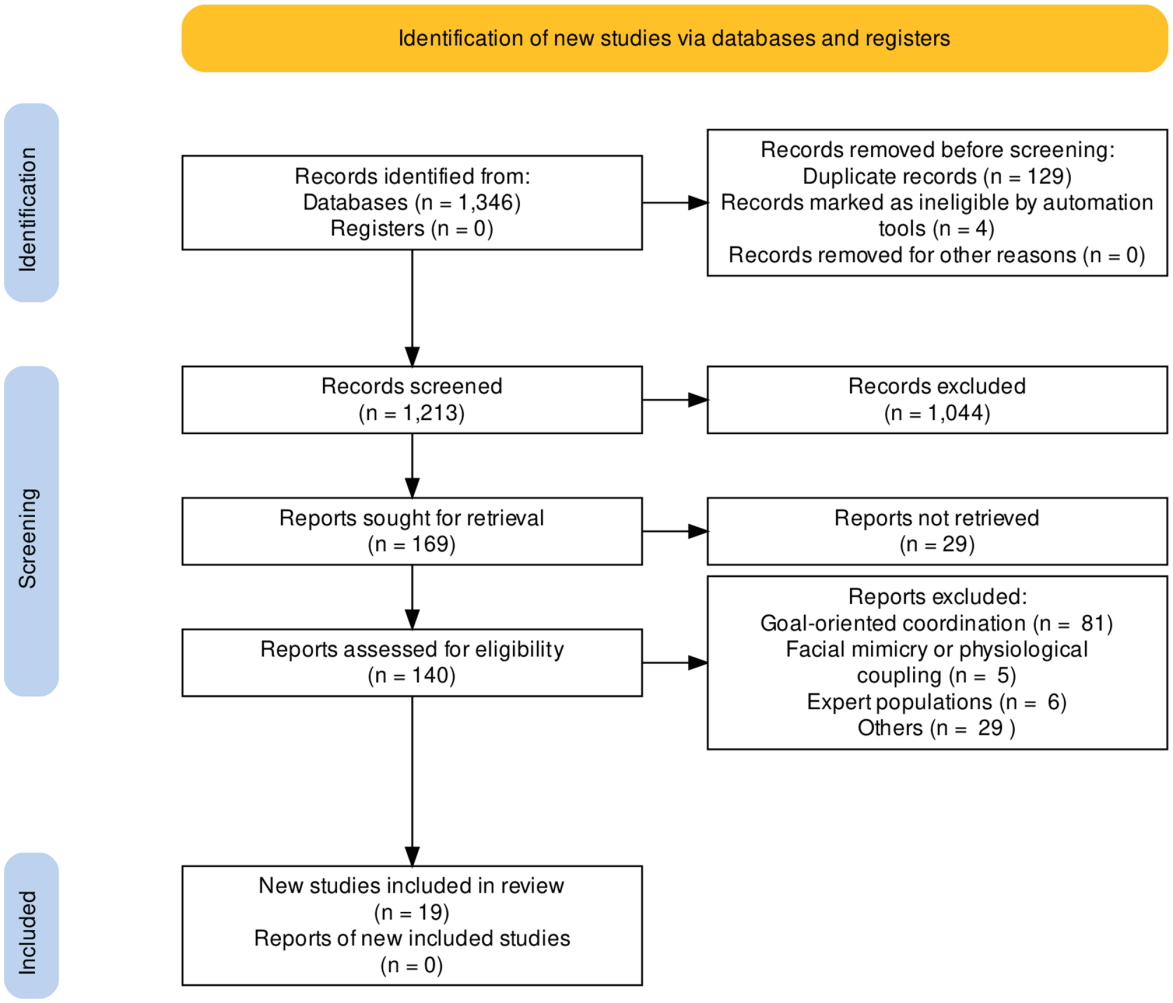
PRISMA flow diagram.

Following the PRISMA protocol (see Figure 3, generated with PRISMA2020, Haddaway and McGuinness, 2020), the 1,213 articles were screened based on the abstracts. Two research team members (the first two authors) independently conducted the screening based on the abstracts. This screening was completed with an inter-rater agreement of 95.8%. A follow-up consensus conference was held that examined the differences, which resulted in minor changes to the wording of the inclusion and exclusion criteria, which have been reflected in this paper. These changes were focused on improving the repeatability of the work by removing ambiguity from the criteria. For example, the initial wording of one of the inclusion criteria indicated that studies should include adult populations. This only implicitly excluded children, and therefore, studies that involved adult–child interactions were not clearly included or excluded. Following these changes to the criteria, the remaining articles all had abstracts where there were minor uncertainties around whether they should be included, for example, using the word “group,” which may or may not involve dyadic interaction. The decision was made that all of these articles should be included in the full-text review for clarification, resulting in an inter-rater agreement of 100% following the conference. A total of 140 articles were selected for full-text review. Articles including facial expressions or gaze (Louwerse et al., 2012; Kruzic et al., 2020), peripheral and neural synchrony (Fusaroli et al., 2016; Varlet et al., 2020) were rejected because we only focus on motor correlates of social interaction. Studies including experimental designs instructing participants to coordinate with each other (Noy et al., 2011; Gueugnon et al., 2016) or to coordinate with an external stimulus (e.g., metronome – see Wu et al., 2013; Mukai et al., 2018) were also rejected because we focus on spontaneous interpersonal coordination. Finally, studies including participants that were friends, romantic partners, or experts (e.g., dancers, musicians) were rejected because we focused on previously unacquainted individuals and non-expert populations that can increase capacity for interpersonal coordination. A final set of 19 articles was retained for the present review (see Supplementary Table S1 in Supplementary Material).

#### Population

Overall, sample sizes ranged from 12 to 192 individuals across the studies. Demographics of the population (e.g., gender, age, sociocultural background) were not always provided (e.g., Nordham et al., 2018), neither were physical properties (e.g., handedness, height or weight). Dyad composition (e.g., same-gender or mixed-gender) was not provided for all studies, and only four studies investigated inter-individual differences such as autistic and personality traits, attachment styles, and emotion regulation abilities (Tschacher et al., 2014, 2018; Cheng et al., 2017; Galbusera et al., 2019).

#### Experimental Paradigms

Experimental paradigms designed to investigate spontaneous interpersonal coordination differed from one study to another and are summarized in the following subsections under the overarching categories of: (i) rhythmic activity and body sway (ii) un/structured conversations, and (iii) problem-solving tasks.

##### Rhythmic Activities and Body Sway

A first set of studies investigated rhythmic activities, such as walking (Cheng et al., 2017, 2020), rocking chairs (Demos et al., 2012), finger flexion (Nordham et al., 2018), “body conversation” (Galbusera et al., 2019), or required participants to stay near each other without any conversation or a common goal (Varlet et al., 2011, 2014).

In Cheng et al.’s studies (2017, 2020), participants walked together on a predetermined pathway while engaged in a conversation or remaining in silence. Participants’ movements were recorded using acceleration sensors attached to their ankles and walking steps coordination evaluated using cross-correlations. In Cheng et al.’s study (2017), participants completed self-reports for autistic traits (Baron-Cohen et al., 2001), revealing that pairs composed of women and individuals with lower autistic traits had a greater tendency to walk in synchrony. In Cheng et al.’s study (2020), participants completed an interpersonal judgment scale (Byrne, 1971), assessing their impressions about other participants, before and after the silent and conversation walk. Results revealed that paired strangers tend to synchronize their footsteps spontaneously, and this synchronization was associated with an increase in their mutual positive impressions. Moreover, a first good impression among walkers facilitated footstep synchronization.

In Demos et al.’s study (2012), participants rocked their chairs while watching a landscape scene with visual (e.g., looking at each other) or auditory (e.g., adding sandpaper under the chair or listening to music) feedback. Magnetic sensors attached to the headrest of each chair recorded participants’ movements, and participants completed self-reports about their subjective feeling of connectedness toward their partner. Cross-correlations computed levels of synchrony, revealing higher levels of synchrony when visual and auditory (e.g., sandpaper under the chair) feedback was provided. Interestingly, while music did not increase levels of synchrony, it was associated with a greater sense of connectedness among participants.

In Nordham et al.’s research (2018), participants flexed their fingers while seeing each other or not, without being instructed to coordinate their movements. A digital goniometer recorded participants’ movements. Frequency differences and Dwell percentage provided an index of interpersonal coordination and its stability over time. Without being instructed to coordinate, participants adjusted their frequency to each other, increasing the time length of interpersonal coordination. This mutual influence remains even after visual feedback (e.g., seeing each other) was removed.

In Galbusera et al.’s research (2019), participants performed a “body conversation task,” conversing using body movements. Kinect cameras recorded participants’ limb movements, and self-reports used to assess their affective state (Breyer and Bluemke, 2016) and self-regulation of affect (Lavender et al., 2017) before and after the body conversation. Cross-correlations computed levels of interpersonal coordination in real and surrogate datasets generated by shuffling limb segment velocity. This procedure provides a baseline for controlling “coincidental” synchrony by distinguishing “pseudo-interactions” from real interactions. While interpersonal coordination was associated with higher reports of positive affect, it also associated with greater difficulties in self-regulation processes. Interestingly, difficulties in self-regulation and fluctuation of positive affect were negatively associated with levels of intrapersonal synchrony.

In Varlet et al.’s study (2011), participants performed an individual visual tracking task near each other. Fifteen reflective markers placed on the right side of each participant were captured by eight infrared cameras for tracking head, torso, and limb movements. Transition frequency provided an index for pattern transition (e.g., in-phase to anti-phase pattern). Cross-wavelet transform computed relative phase values, providing information about frequency synchronization between two time series. Spontaneous interpersonal postural coordination was observed between pairs of participants, influencing head movements and intrapersonal ankle–hip coordination.

In Varlet et al.’s research (2014), participants were standing back-to-back or in front of each other on a ship in motion, while accelerometers tracked body sways. Detrended fluctuation analyses determined statistical self-affinity of time-series and cross-spectral coherence provided power transfer from one signal to another. Despite the strong influence of ship motion on postural activity, visual contact between participants increased interpersonal coordination and influenced intrapersonal body sway.

##### Structured and Unstructured Conversations

A second set of studies observed the emergence of interpersonal coordination during structured or unstructured conversations using monologue or free conversations (Fujiwara and Daibo, 2016; Fujiwara et al., 2019), cooperative or competitive framing (Paxton and Dale, 2013, 2017; Tschacher et al., 2014, 2018; Lozza et al., 2018), or pre-scripted and turn-taking settings (Schmidt et al., 2012; Hale et al., 2020).

In Fujiwara and Daibo (2016) and Fujiwara et al.’s studies (2019), participants were engaged in monologues or free conversations. Video-recording combined with motion-energy analysis (Fujiwara et al., 2019), or video image analysis software (Fujiwara and Daibo, 2016) extracted participants’ movements. Cross-wavelet coherence comparing time and frequency domains provided indices of interpersonal coordination. Coherence measured the similarity between two time series, while wavelet transform methods extracted synchronization patterns (e.g., in-phase or anti-phase). In Fujiwara and Daibo’s study (2016), these methods were used to compare real and virtual pairs generated by shuffling original time series. As expected, real pairs displayed higher levels of synchrony than virtual pairs. In Fujiwara et al.’s research (2019), similar methods were used to compare same-gender dyads. While pairs of women displayed consistently higher coherence, the effect of gender on synchronization patterns remains inconclusive, despite a trend for anti-phase patterns in dyads composed of men.

In Lozza et al.’s research (2018), participants were engaged in competitive role-play (e.g., negotiating a company car). Video-recording combined with motion energy analysis extracted participants’ movements. Additionally, two independent judges rated video-recordings using a joystick tracking method with affiliation (e.g., hostile vs. friendly) on the x-axis and dominance (e.g., dominant versus submissive) on the y-axis. Cross-correlations computed indices of interpersonal coordination based on participants’ movements and surrogate datasets, while affiliation and dominance scores were averaged between judge ratings. Negotiation outcomes were neither predicted by interpersonal coordination nor affiliation or dominance, although higher levels of synchrony were observed in real compared to virtual pairs. However, exchange durations were influenced by complementary roles. Dyads with complementary profiles (e.g., submission combined with dominance) were quicker to reach an agreement compared to similar profiles (e.g., dominance and dominance).

In Paxton and Dale’s studies (2013, 2017), participants conducted argumentative (e.g., convincing each other) and affiliative (e.g., discussing mutually enjoyed media) exchanges based on participants’ opinions. While in the first study (Paxton and Dale, 2013), video-recording combined with frame-differencing method was used for extracting participants’ movements, the second study used Google Glass for tracking participants’ head movements while their vision was occulted by a red filter (Paxton and Dale, 2017). Indices of interpersonal coordination were computed using cross-correlations (Paxton and Dale, 2013) or cross-recurrence quantification analysis for computing cross-recurrences between two time series (Paxton and Dale, 2017) and compared to surrogate datasets. In Paxton and Dale’s study (2013), argumentative exchanges decreased the amount of synchrony significantly but no associations were observed with the affective state. A similar pattern was observed in Paxton and Dale’s study (2017) where argumentative context decreased head synchrony. More precisely, synchrony during friendly conversations was indistinguishable from chance, while synchrony during argumentative context was significantly lower than what would be expected by chance. Interestingly, the red filter increased the turn-taking pattern of head movements when presented as a dual task (e.g., remembering the number of times it appeared). However, it did not affect head movement patterns when presented as noise (e.g., bug with Google Glass).

In Tschacher et al.’s research (2014, 2018), participants engaged in competitive (e.g., convincing each other) or cooperative conversation (e.g., developing a shared position) using a prescripted list of arguments. In both studies, video-recording and motion-energy analyses extracted participants’ movements. Additionally, in Tschacher et al.’s study (2018), participants completed self-reports of Big Five dimensions of personality (Borkenau and Ostendorf, 1991), interpersonal functioning (Horowitz et al., 1994), attachment quality (Carver, 1997) and empathy (Davis, 1983). In both studies, cross-correlations were used to compute indices of interpersonal coordination. In Tschacher et al.’s study (2018), “social present” was associated with the length of synchronized interaction and considered an index of mutual awareness during social interactions. In Tschacher et al.’s study (2014), higher levels of synchronization were observed during competitive conversations, associated with emotional arousal. Competitive settings were also associated with prolonged length of synchronization, especially for dyads composed of men or participants scoring higher on Openness to experience, avoidant attachment and lower narcissistic interpersonal style. Interestingly, these conversations were considered less constructive in the participants’ assessments.

In Hale et al.’s study (2020), participants described pictures during 16 trials using a turn-taking pattern, alternating monologues and dialogues. Magnetic motion tracking devices attached to the forehead and upper back recorded participants’ movements. Indexes of interpersonal coordination were computed using cross-wavelet coherence in real and virtual pairs. Synchronization of head movements in low frequencies (0.2–1.1Hz) were associated with a time lag of 600ms, suggesting an automatic reactive process underlying behavioral mimicry without anticipatory mechanisms. On the other hand, synchronization in high frequencies (2.6–6.5Hz) displayed synchrony lower than chance, related to fast nodding associated with active listening during turn-taking conversations.

Finally, Schmidt et al. (2012) asked participants to enact knock-knock jokes. Video-recording and magnetic motion tracking were used for tracking participants’ head and wrist movements. Notably, video-recordings were also coded manually by four external raters, while indices of interpersonal coordination from trackers were extracted using cross-spectral analyses to determine the relationship between two time series as a function of frequency and compared to surrogate datasets. Although the experimental design involved a turn-taking setting, interpersonal coordination rose above chance during these exchanges and displayed an in-phase pattern.

##### Collaborative Problem-Solving Tasks

A final set of studies used collaborative problem-solving tasks, such as building towers (Abney et al., 2015), collaborative decision-making (Wiltshire et al., 2019), and an idea generation task (Sun et al., 2019).

In Abney et al.’s study (2015), participants built a tower using marshmallows and uncooked spaghetti. Video-recording combined with the frame-differencing method was used for movement tracking. Participants completed self-reports about their perceived dominance/passivity during the task. Cross-recurrence quantification analyses were computed on real and surrogate datasets for computing indexes of interpersonal coordination. Task performance assessed by tower height was positively associated with lower levels of coordination. This result suggests that loosely coupling (e.g., reduced interpersonal coordination) rather than higher levels of coordination improves collective performance during a problem-solving task.

In Wiltshire et al.’s study (2019), participants performed the moon base alpha task, a computer simulation where participants need to collaborate to restore oxygen to the settlement. Participants were video-recorded, and a frame-differencing method was used for extracting participants’ movements. Surrogate datasets and cross-wavelet coherence were computed to assess interpersonal coordination. While in-phase coordination was associated with better task performance, dyads exhibiting stable degrees of movement coordination over the duration of the task performed better than those that had a sharp decrease in their coordination.

Finally, Sun et al. (2019) used a virtual reality setting, manipulating participants’ avatars (e.g., humanoid versus abstract cube), while participants were engaged in an idea generation task in cooperative (e.g., generating ideas together) or competitive framing (e.g., competing against each other). The Oculus Rift head-mounted display and hand trackers were used to record participants’ head and hand movements. Cross-correlation and surrogate datasets were used to compute levels of interpersonal coordination. Head synchrony was associated with social closeness, while competitive framing increased turn-taking behaviors. Interestingly, participants’ avatars did neither influence levels of synchrony nor social presence in collaborative or competitive conditions.

## Discussion

The present systematic review aimed to investigate the last decades of research on spontaneous interpersonal coordination, following several calls for adopting a “two-body approach,” encouraging new experimental paradigms assessing the mechanisms underlying “real” and “live” social interaction (Hari and Kujala, 2009; Dumas, 2011; Schilbach et al., 2013). Results of the present review testify that this call was indeed well-received considering the increase in studies investigating spontaneous interpersonal coordination in ecological settings. Furthermore, exploratory analyses revealed a sharp increase of scientific publications associated with interpersonal coordination, with the development of new techniques based on computational analyses using low-cost methods for kinematic recordings, reducing financial and temporal burden, previously encountered with expansive recording settings and manual coding methods. The increasing varieties of settings used in this research field have improved our understanding of spontaneous interpersonal coordination mechanisms. Nevertheless, it is also worth noting that this results in higher fragmentation of theoretical assumptions, methodology, and outcomes, rendering difficult the emergence of a clear and coherent picture.

### The Problem of Terminology

The exploratory research revealed the widespread use of interpersonal coordination as a terminology encompassing broad research topics. It remains unclear whether the sharp increase of publications in this area is due to the genuine accumulation of studies investigating interpersonal coordination as a motor correlate of social interaction or as a linguistic artefact resulting from the overinclusive use of this terminology. Automatic algorithms for keyword extractions failed to provide a meaningful set of keywords, reflecting the inadequacy of the sole interpersonal coordination terminology to delineate a precise research topic. While this lack of efficiency could be attributed to the current caveats in keyword processing in scientific databases (Shah et al., 2003), this also reflects the many research topics embedded within the framework of dynamical systems theories. In that respect, the outcomes provided by the LDA algorithm illustrated the various domains where interpersonal coordination is applied. From mother–infant interaction in developmental psychology to clinical conditions (e.g., autism, schizophrenia) and neurophysiological phenomena at the intra-individual level (e.g., neural synchrony) to larger dynamics processes (e.g., collective sport), interpersonal coordination encompasses more or less all types of social interactions. Therefore, considering its widespread applications, it is not surprising to observe a proliferation of various terminologies associated with interpersonal coordination.

### Jingle–Jangle Fallacy

Although this was not always the case, most of the studies presented in this systematic review provided a working definition of interpersonal coordination; however, the wording differed from one study to another. A first set of studies used the terminology *interpersonal coordination* referring to “dynamical process of self-organization,” “spatiotemporal organization of movement,” or “dynamical entrainment” (Varlet et al., 2011, 2014; Demos et al., 2012; Fujiwara et al., 2016; Paxton and Dale, 2017; Nordham et al., 2018; Fujiwara et al., 2019; Wiltshire et al., 2019). On the other hand, others used the terminology *synchrony*, referring to “variables of a system becoming entrained” or as “specious present,” and “smooth meshing in time of the simultaneous rhythmic activity” or “the coordinated overall body movement” of two interacting individuals (Schmidt et al., 2012; Paxton and Dale, 2013; Tschacher et al., 2014, 2018; Abney et al., 2015; Cheng et al., 2017, 2020; Lozza et al., 2018; Galbusera et al., 2019; Sun et al., 2019). Finally, Hale et al. (2020) referred to mimicry and complementary behavior. Several reports acknowledged the overgrowing vocabulary used in this research field (Paxton and Dale, 2017; Lozza et al., 2018), providing a list of the terminologies used within the literature: accommodation, alignment, the “chameleon effect,” complementarity, contagion, coordination, coupling, mimicry, synchrony, synergy, reciprocity. Additionally, Paxton and Dale (2017) provided a distinction between *coordination* (or coherence) and *synchrony*, the former referring to “the idea that individuals affect one another’s behavior over time as a result of their interaction,” the latter to specific cases of coordination when individuals “exhibit the same behavior at the same time” (Paxton and Dale, 2017, p. 2). On the other hand, Cheng et al. (2017, 2020) demarcated *implicit synchrony*, when actors do not share the same goal, from *explicit synchrony* as a result of intentional motor coordination or joint action. Hale et al. (2020) attempted to distinguish between *mimicry*, referring to “events where two people perform the same action” and *complementary behavior*, when two people perform different actions, echoing the definition provided by Bernieri and Rosenthal (1991). Finally, Varlet et al. (2011, 2014) were interested in differentiating mechanisms involved in intra- and interpersonal limbs movements. This effort to delineate these different concepts echoes other attempts within the literature (Dumas and Fairhurst, 2019). Nevertheless, these current coexisting labels lead to a jingle–jangle fallacy, as it becomes unclear whether the literature is referring to different phenomena using identical labels (jingle) or describe the same processes but labeled differently (jangle). For example, a large amount of the literature refers to interpersonal coordination without clearly delineating implicit and explicit coordination that renders it difficult to disentangle automatic from goal-oriented processes. On the other hand, synchrony and coordination are often conflated and used as synonyms. Consequently, while some use synchrony in the specific context of in-phase coordination (Paxton and Dale, 2017), others do not differentiate synchrony from coordination without delineating in-phase versus anti-phase coordination (Tschacher et al., 2014, 2018).

### From Embodied Theories to Coordination Dynamics

These conflated constructs between synchrony and coordination appear to spring from two theoretical streams, studying interpersonal coordination from different standpoints. On the one side, embodied cognition theories postulate that cognitive processes arise from sensorimotor experiences (Barsalou, 2010). Here, interpersonal coordination is understood as an expression of cognitive processes grounded in brain–body interactions such as language (Gallese, 2008), empathy (Goldman and de Vignemont, 2009), or self-awareness (Borghi and Cimatti, 2010). While embodied cognition theories acknowledge the role played by dynamical exchanges occurring between the environment and the cognitive systems (Barsalou, 1999), this theoretical framework remains wired to individual and separate bodies. On the other side, dynamical system theories (Kelso, 1995) consider interpersonal coordination as a self-organization process that emerges from two biological systems interacting. Here, interpersonal coordination is conceived as “groundless,” in line with the enactive approach suggested by Varela et al. (1991); for a recent discussion on “groundless,” see Meling, 2021. Applied to biological and physical phenomena, this theoretical model has been expanded to interpersonal coordination, understood here as dynamical patterns influenced by attractors. As formulated by Haken et al. (1985) in their model of limb coordination, two main types of attractors co-exist: the 0° or in-phase mode where two systems are symmetrical and the 180° or anti-phase mode, where the two systems are in alternate phases. Outside of these two attractors, greater fluctuations and deviations are observed in motor coordination. Thus, according to this theoretical framework, interpersonal coordination is a by-product of physical law, going beyond cultural conventions. Consequently, interpersonal coordination emerges from perceptual and social influences, where one can distinguish “soft” (e.g., gaze exchanges and social gestures) from “hard” constraints (e.g., ship mechanical motion) that impact its pattern and stability (Varlet et al., 2014). This distinction partly echoes the definition provided by Athreya et al. (2014) between mechanical and perceptual constraints and is reflected in the present systematic review.

### Soft and Hard Constraints

Grounded in dynamical system theories, Varlet et al. (2011, 2014), Demos et al. (2012), and Nordham et al. (2018) investigated the influence of perceptual information exchanges between participants while standing in front of each other or conducting rhythmic activities such as rocking chairs or finger flexions. While participants were not instructed to synchronize their movements, all of these studies observed spontaneous entrainment of body movements between individuals and amplified when participants shared perceptual information such as visual or auditory feedback. These results stress the fact that sole sensorial information exchanges lead to spontaneous coordination. Notably, these effects are also observed during goal-oriented rhythmic activities in a dyadic and larger ensemble (Miyata et al., 2017; Zhang et al., 2019) and can last after perceptual information is removed, leading to a form of “motor entanglement” between individuals (Ikegami and Ganesh, 2014; Soliman et al., 2015). Schmidt et al. (2012) suggested that the dynamical patterns identified in lab-controlled environments during rhythmic activities are similar to those arising in more ecological settings such as casual conversations. This implies that social exchanges could be investigated using similar methods used in rhythmic activities, opening new research opportunities. On the other hand, grounded in embodied cognition theories, another stream of studies investigated the influence of inter-individual differences in interpersonal coordination. For example, Cheng et al. (2017) reported lower tendencies for interpersonal coordination for individuals with higher scores on the autistic quotient (Baron-Cohen et al., 2001). This observation is consistent with previous findings observing a disruption of spontaneous interpersonal coordination in clinical populations, such as individuals with schizophrenia (Varlet et al., 2012; Kupper et al., 2015), social anxiety (Heerey and Kring, 2007; Varlet et al., 2014), depression (Breznitz and Sherman, 1997; Wiltshire et al., 2020), and autism (Marsh et al., 2013; Georgescu et al., 2020). These clinical conditions are often associated with social cognition impairments, suggesting shared mechanisms between joint action and joint cognition (Humphreys and Bedford, 2011). Additionally, Cheng et al. (2017) observed a greater tendency for interpersonal coordination in women, echoing the pattern observed by Fujiwara et al. (2019), suggesting a role of pro-sociality and interpersonal sensitivity in interpersonal coordination, usually associated with the female gender. On the other hand, male dyads displayed a greater tendency for anti-phase patterns (Fujiwara et al., 2019) and an extended period of synchrony (Tschacher et al., 2018). However, this pattern remains unexplained in both studies, requiring further investigations. Using conversational settings, Hale et al. (2020) and Schmidt et al. (2012) stress the impact of turn-taking behavior on interpersonal coordination patterns. Sun et al. (2019) also observed this pattern in virtual reality, reporting an increase of variance in synchrony scores due to turn-taking behavior. Thus, conversations allow interpersonal coordination to arise despite their difference from rhythmic activities, although its content modulates interpersonal coordination. In that respect, Paxton and Dale (2013, 2017) observed a decrease in motor synchrony during argumentative exchange compared to friendly conversation. On the other hand, Tschacher et al. (2014) and Lozza et al. (2018) observed the opposite pattern, where competitive conversations increased non-verbal synchrony. These mixed findings might be due to different experimental settings. On the one hand, Paxton and Dale (2013, 2017) used prompts from a list of topics where participants expressed their “real” disagreement beforehand, therefore promoting self-differentiation. On the other hand, Tschacher et al. (2014) and Lozza et al. (2018) framed their experimental task in terms of role-playing where participants’ discourses were pre-scripted. Thus, this subtle difference in the experimental settings might have changed participants’ experience of the task, perceived as a game rather than an actual argument. Sustaining this idea, Tschacher et al. (2014) observed an increase of positive and negative affect during competitive rather than cooperative conditions, suggesting a role of emotional arousal in eliciting interpersonal coordination. Taken together, these findings highlight how subtle contextual changes can drastically change the outcomes associated with interpersonal coordination.

### An Elusive Marker of Social Interaction

The expansion from highly controlled settings investigating perceptual influences to ecological settings manipulating social contexts unravels the richness and complexity of interpersonal coordination and questions its interpretation as a marker of social affiliation. Studies investigating perceptual influences (Varlet et al., 2011, 2014; Demos et al., 2012; Nordham et al., 2018) revealed that the simple sharing of sensorimotor information leads to spontaneous interpersonal coordination. Notably, these studies have considered physical properties such as height and weight, often neglected by studies investigating social influence. According to dynamical system theories, it is not surprising that two systems sharing similar physical properties are more prone to display interpersonal coordination as the degrees of freedom between these two systems are already reduced (Vallacher et al., 2005; Słowiński et al., 2016). This physical law stresses the importance of generating surrogate datasets to distinguish between arbitrary interpersonal coordination arising in virtual pairs from spontaneous coordination between two individuals interacting. While most studies adopt this practice, this is not always the case, and results should be interpreted with caution. Nevertheless, in all studies, incidental interpersonal coordination arose and was often associated with indices of social connection. Cheng et al. (2020) observed an increase of mutual impression between strangers after walking together, while Sun et al. (2019) observed a positive association between head synchrony and social closeness. On the other hand, Demos et al. (2012) stressed the role of music to elicit a sense of shared experience that might mediate the effect of synchrony on connectedness. This result is not surprising considering that previous studies demonstrated the potential evolutionary role of music and dance for social bonding (Dunbar, 2012). Interpersonal coordination was also associated with positive affect (Tschacher et al., 2014, 2018). However, Galbusera et al. (2019) noted that this increase in positive affect was also associated with greater difficulties in emotion regulation abilities, suggesting a potential detrimental effect of interpersonal coordination on self-regulation processes. This observation is important as preexisting literature has often emphasized positive aspects of interpersonal coordination, suggesting the lack of coordination as a marker of clinical conditions.

### From Synchrony to Meta-Stability

The tendency to consider interpersonal coordination as an optimal solution, also labeled as the “more is better” hypothesis by Abney et al. (2015), is challenged by recent findings highlighting a more contrasted picture. Abney et al. (2015) observed a negative association between interpersonal coordination and dyadic performances in problem-solving, while Wiltshire et al. (2019) noted that the stability of coordination over time appears as a better predictor than peaks of synchrony. These observations echo those from Lozza et al. (2018), observing a U-shape relationship between non-verbal synchrony and relationship quality, suggesting that loose coupling appears as a better indicator of collective performances. These findings are similar to observations from developmental studies, stressing that motor synchrony does not necessarily reflect a healthy form of attachment by preventing the infant from building self-awareness (Maister et al., 2020). Mother–infant interactions are characterized by desynchronization and mismatch, which are crucial to building social skills and self-other distinction (Tronick and Cohn, 1989). Tschacher et al. (2018) observed that attachment styles inherited from infancy still modulate non-verbal synchrony duration in adulthood. Consequently, extended periods of synchrony can be interpreted either as openness to mutual exchange or as avoidant tendencies associated with perceived complex and demanding interactions. These contrasting explanations highlight the versatility of interpersonal coordination and its difficult interpretation as a marker of social interaction without considering its association with social norms and task constraints. The positive outcomes usually associated with interpersonal coordination can quickly backfire (e.g., Galbusera et al., 2019). This switch from beneficial to detrimental outcomes can also be explained by social norms. Dalton et al. (2010) observed self-regulation impairments when individuals were mimicked by someone from a higher social status or out-group member. Additionally, Wood et al. (2018) observed that interpersonal coordination impairs group performance during coordination tasks through increasing group conflict. These results imply that group coordination tasks require a hierarchical structure disrupted by synchrony, making it impossible to take complementary and turn-taking exchanges. These observations reflect the concept of meta-stability, defined as the ability to be “in and out” of synchrony, where phase transition rather than in-phase or anti-phase synchronization appears as a better signature of behavioral flexibility (Tognoli and Kelso, 2014; Mayo and Gordon, 2020). Therefore, statistical analyses based on cross-correlations, a method widely used in the literature (Tschacher et al., 2014, 2018; Cheng et al., 2017, 2020; Lozza et al., 2018; Galbusera et al., 2019; Sun et al., 2019), appear insufficient to capture this phenomenon. On the other hand, spectral analyses such as cross-wavelet or cross-recurrence quantification analysis might offer a more detailed capture of the dynamical phenomena taking place during social exchanges. This was illustrated by Hale et al. (2020) and Paxton and Dale (2017) using costly experimental settings, while Fujiwara et al. (2016, 2019), Wiltshire et al. (2019), and Abney et al. (2015) were able to compute such analyses using simple video-recordings. During the last decade, the development of new methods using low-cost equipment such as frame-differencing (Paxton and Dale, 2013), motion energy analysis (Ramseyer, 2020), Kinect camera (Papadopoulos, 2014), or virtual agents (Bailenson et al., 2008; Dumas et al., 2014) offered new ways of studying interpersonal coordination, improving our understanding of this phenomenon, but also rendering it difficult to obtain a coherent picture.

## Conclusion

To conclude, interpersonal coordination is a flourishing research area that has gathered considerable attention during the last decade due to a theoretical shift in social neurosciences from studying intra-individual to inter-individual processes. New methods providing low-cost solutions for movement recording and analyses are part of the development of this research field. Illustrating its vibrancy, the present review offers a glimpse of the experimental methods currently used to study spontaneous interpersonal coordination and unravel its mechanisms. While the preexisting literature has often emphasized the role played by interpersonal coordination as a social glue, these new findings highlighted a more nuanced picture, questioning the nature of interpersonal coordination and our understanding of its meaning. Different theoretical accounts of interpersonal coordination have delivered inconsistent definitions, resulting in an overgrowing terminology that renders it difficult to understand what interpersonal coordination is exactly. From one side, dynamical system theories highlighted the roles of perceptual and contextual constraints influencing the strength of the coupling between individuals, where interpersonal coordination is understood as a pattern arising from dynamical self-organization processes. On the other hand, studies grounded in embodied cognition theories stressed the role of inter-individual differences, where interpersonal coordination is understood as a signature of intra-individual neuropsychological processes. Recent attempts to merge these visions have already been made studying interpersonal coordination impairments in clinical conditions, such as schizophrenia (Varlet et al., 2012; Kupper et al., 2015) or autism (Marsh et al., 2013; Georgescu et al., 2020). Signal processing based on spectral analysis might also offer promising insights into this complex phenomenon by comparing time and frequency domains. Considering social interaction in a broad sense of information exchanges between individuals, it is important to acknowledge that this review did not present an exhaustive examination of the whole literature on interpersonal coordination and its association with social interaction. Interpersonal coordination is indeed at the crossroad between several research topics ranging from early social interactions (Feldman, 2007) to joint action in laboratory-based experiments (Sebanz and Knoblich, 2021) or more ecological contexts such as sport (Zumeta et al., 2016) or aesthetic experiences (Vuoskoski and Reynolds, 2019). Therefore, it is important to consider this review as a snapshot of this emerging research field that has evolved and still continues to grow rapidly for a decade. Consequently, one of the main challenges for the interpersonal coordination research field will be to build bridges across scattered research fields and open a dialogue between different theoretical frameworks to provide a more ecological and holistic understanding of the motor correlates of social interaction.

## Data Availability Statement

The datasets presented in this study can be found in online repositories. The names of the repository/repositories and accession number(s) can be found at: Open Science Framework https://osf.io/x9cb6/.

## Author Contributions

JA conducted the exploratory research and the systematic review and wrote the manuscript. AC conducted the screening of the articles and revised the manuscript. SM, DK, DR, and AS participated in the keywords selection and revised the manuscript. NH supervised the whole process and revised the manuscript. All authors contributed to the article and approved the submitted version.

## Funding

This research project is supported by the Doctoral Alliance Training co-funded by the European Union’s Horizon 2020 research and innovation programme under the Marie Skłodowska-Curie grant agreement [801604].

## Conflict of Interest

The authors declare that the research was conducted in the absence of any commercial or financial relationships that could be construed as a potential conflict of interest.

## Publisher’s Note

All claims expressed in this article are solely those of the authors and do not necessarily represent those of their affiliated organizations, or those of the publisher, the editors and the reviewers. Any product that may be evaluated in this article, or claim that may be made by its manufacturer, is not guaranteed or endorsed by the publisher.

## Footnotes

^1^ https://osf.io/x9cb6/
